# Ozurdex (dexamethasone intravitreal implant) for the treatment of intermediate, posterior, and panuveitis: a systematic review of the current evidence

**DOI:** 10.1186/s12348-019-0189-4

**Published:** 2020-01-10

**Authors:** Saanwalshah Samir Saincher, Chloe Gottlieb

**Affiliations:** 1 Department of Health-Sciences, Bristol Medical School, University of Bristol, First Floor, 5 Tyndall Avenue, Bristol, BS8 1UD UK; 2 The Ottawa Hospital Research Institute, Ottawa, Ontario Canada; 3 University of Ottawa Eye Institute, The Ottawa Hospital, General Campus, 501 Smyth Road, CCW Box 307, Ottawa, Ontario K1H 8L6 Canada

**Keywords:** Uveitis, Noninfectious uveitis, Intermediate, Posterior, Panuveitis, Ozurdex, Dexamethasone, Intravitreal implant, Macular edema

## Abstract

**Background:**

This study aims to determine if the intravitreal dexamethasone implant (DEX implant, Ozurdex; Allergan, Inc., Irvine, California) is effective for treating intermediate, posterior, and panuveitis as a monotherapy or adjunctive treatment to systemic immunomodulatory therapies.

**Methods:**

A systematic review using MEDLINE, EMBASE, and PubMed database searches was conducted with the Oxford Centre for Evidence-based Medicine Levels of Evidence criteria to select publications. Available background information and patient data from each study was tabulated. Outcomes studied were central retinal thickness (CRT), best corrected visual acuity, intraocular inflammation (anterior chamber cells, vitreous haze), number of patients with prior and concomitant immunomodulatory treatments, intraocular pressure (IOP) elevation (≥ 25 mmHg), and other adverse effects associated with the implant.

**Results:**

One hundred ninety-five (61.51%) patients had previous immunomodulatory treatment while 232 (64.8%) were treated with concomitant immunomodulatory therapy with the DEX implant. CRT decreased by an average of 198.65 μm (42.74%). Visual acuity improved to an average of 0.451 (logMAR) or 20/57 (Snellen) which is a 43.11% improvement from baseline. One hundred seventy-three (59%) of eyes were quiescent at the end of the trials, of which 40 (13.7%) previously inflamed eyes became quiescent. Elevated IOP occurred in 91 (20.6%). The most common adverse events were cataract/posterior subcapsular opacities in 47 (11.03%) patients and conjunctival hemorrhage in 24 (5.44%) patients.

**Conclusions:**

The DEX implant is an effective medication for the treatment of posterior segment uveitis, uveitic macular edema, and results in improved visual acuity. Development of elevated IOP and cataract should be closely monitored as they are tangible risks associated with the DEX implant. This study was not able to determine whether the DEX implant was more effective as a monotherapy or as an adjunctive therapy to systemic immunomodulatory treatment.

## Background

The development of an intravitreal dexamethasone implant (DEX implant Ozurdex; Allergan, Inc., Irvine, California) placed in the eye by an office-based intravitreal injection has changed the landscape of uveitis treatment. With a capsule composed of polymers of lactic acid and glycolic acid, the DEX implant is biodegradable, ensuring that the corticosteroid delivery is localized to the affected eye and a small amount enters the systemic circulation [1]. The DEX implant has been shown to be effective for up to 6 months and reduces the need for repeated periocular or intravitreal corticosteroid injections [1].

While proven to be effective for treating macular edema associated with central retinal vein occlusion and diabetic macular edema, the effects of the DEX implant for treatment of intermediate, posterior, and panuveitis uveitis are not well established in the literature. There is only one prospective cohort study (conducted by Bansal et al.) and one retrospective study (the HURON trial) analyzing the use of Ozurdex for posterior segment inflammation [1, 2]. Therefore, retrospective studies form the base of reports that assess the efficacy and safety of Ozurdex. Despite a lack of prospective trials, the DEX implant has been approved for treating posterior segment inflammation by the US Food and Drug Administration (FDA) as of September 25, 2010, and the National Institute for Health and Care Excellence (NICE) since July 26, 2017 [3, 4]. What remains to be clarified is the effectiveness of the DEX implant as a monotherapy versus adjunct therapy for posterior uveitis, as all studies to date report data from patients with previous or ongoing systemic corticosteroid and/or immunomodulatory treatment in combination with the DEX implant.

In this review, we assess the current literature and report on the effectiveness and safety of the DEX implant for treatment of posterior uveitis, with a focus on the DEX implant as a monotherapy or when used in combination with systemic immunosuppressant therapies.

## Methods

**Fig. 1:**
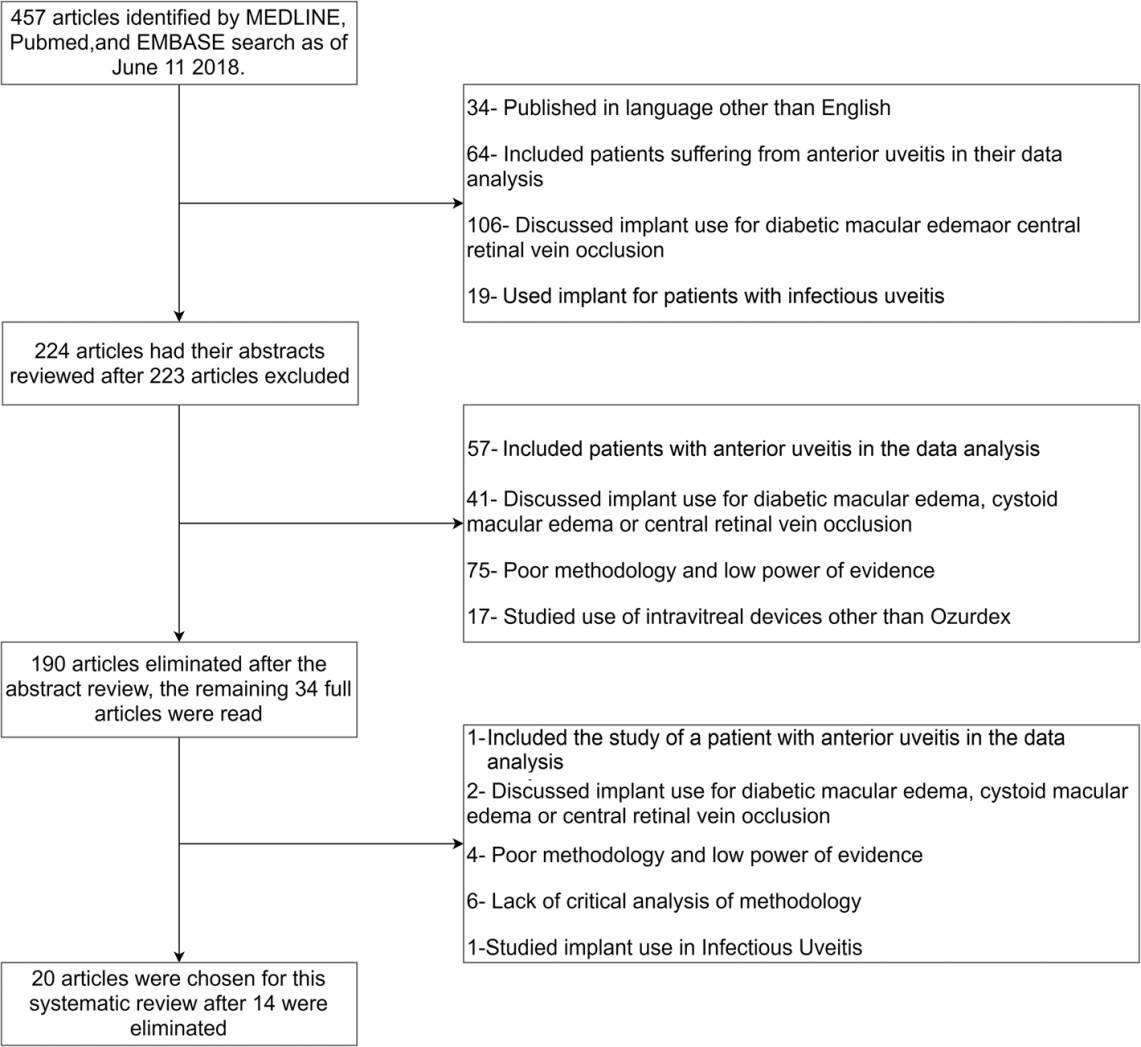
The process of choosing the appropriate articles for this review

The MEDLINE, Embase, and PubMed databases were used to identify articles; the search terms used were “the DEX implant,” “dexamethasone intravitreal implant,” “intermediate uveitis,” “posterior uveitis,” and “panuveitis.” The search was updated periodically until February 2019. Publications reporting the use of the DEX implant for the treatment of non-infectious posterior segment inflammatory disease were included. Figure 1 delineates our search process. The initial search revealed 457 articles, and the following exclusions were reduced to 224. Many studies discussed the use of the DEX implant for treating macular edema or included subjects with anterior uveitis. As they are not in the remit of this study, they were not included in the analysis. After a review of the abstracts of the remaining articles, the search was narrowed to 34 articles. Articles with poor methodology, poor power of evidence, expert opinion, or lacking explicit critical analysis according to the Oxford Centre for Evidence-based Medicine Levels of Evidence criteria (OCEMLEC) were excluded [5]. Following the above exclusions, 20 of the 34 articles were selected for full analysis. The Oxford Centre for Evidence-based Medicine Levels of Evidence criteria allowed for the inclusion of articles that ranked highly, such as randomized clinical studies and other systematic reviews with cohort studies [5]. Figure 1 summarizes the methods for article selection.

The total number of eyes, number of patients, and the average age of patients across all trials was tabulated. The population group (pediatric/adult) and the type of each study were also recorded. Available patient data on central retinal thickness (CRT), visual acuity, anterior chamber and vitreous cells, intraocular pressure (IOP), and adverse effects were tabulated from each report. The number of patients with ongoing or previous systemic immunomodulatory treatments was also tabulated.

When analyzing the article by Lowder et al., only the results from the 0.7 mg DEX implant group were studied, as this is the dose presently available from the manufacturer.

### Central retinal thickness (CRT)

Some studies included CRT measurements from optical coherence tomography (OCT). Available CRT measurements before and after the DEX implant were tabulated. The change in micrometers and percent change were tabulated for each study and averaged across all studies. The articles by Jaffe et al., Bratton et al., Ragam et al., Myung et al., and Habot-Wilner et al. did not measure central retinal thickness.

### Visual acuity

The visual acuity before the DEX implant and the last reported visual acuity in the study were tabulated and converted to the logMAR scale. The numerical change and percent change were calculated and averaged across all studies. The study by Lowder et al. did not report a baseline or final visual acuity and instead reported an average 10.8 letters of improvement in the 0.7 mg DEX implant group. Similarly, the case study by Arcinue et al. also did not measure baseline or final visual acuity and only mentioned that their subjects improved from baseline. Because these two papers did not include specific visual acuity measurements, they were excluded from our calculations.

### Inflammatory markers

The inflammatory markers measured were anterior chamber cells and vitreous haze. The number of quiescent eyes at the start and end of each study was tabulated. Quiescence was defined as an anterior chamber cell score of 0.5 or less and/or a vitreous haze score of 1 or less. Lam et al. reported that they could not measure vitreous haze in their study because it was not documented. The studies by Adan et al., Miserocchi et al., Ryder et al., Bansal et al., Lei and Lam., Taylor et al., Ragam et al., and Arcinue et al. (8/20 studies) did not report data on inflammatory markers and were excluded from our calculations.

### Adverse events (AEs)

The adverse events associated with the DEX implant in each study were logged. Adverse events were expected or unexpected events such as cataract, elevated IOP, conjunctival hemorrhage, and floaters. The period prevalence for each adverse event from all studies was independently calculated, and then, an average across all studies was calculated. The period is defined as the duration of each study. The study by Bratton et al. was excluded from the analysis of posterior subcapsular opacities (PSCOs) and anterior chamber migration because the study did not provide any quantitative data on these adverse events.

### Intraocular pressure (IOP)

Elevation of a patient’s IOP ≥ 25 mmHg at any point in a study was defined as an IOP adverse event. The number of IOP adverse events and period prevalence was tabulated. Data was able to be retrieved from all studies.

### Systemic immunomodulatory treatment

The number of patients with immunomodulatory treatments preceding the DEX implant and number of patients with concomitant systemic immunomodulatory treatments were tabulated. Oral corticosteroid was included as an immunomodulatory treatment.

## Results

### Central retinal thickness

**Fig. 2:**
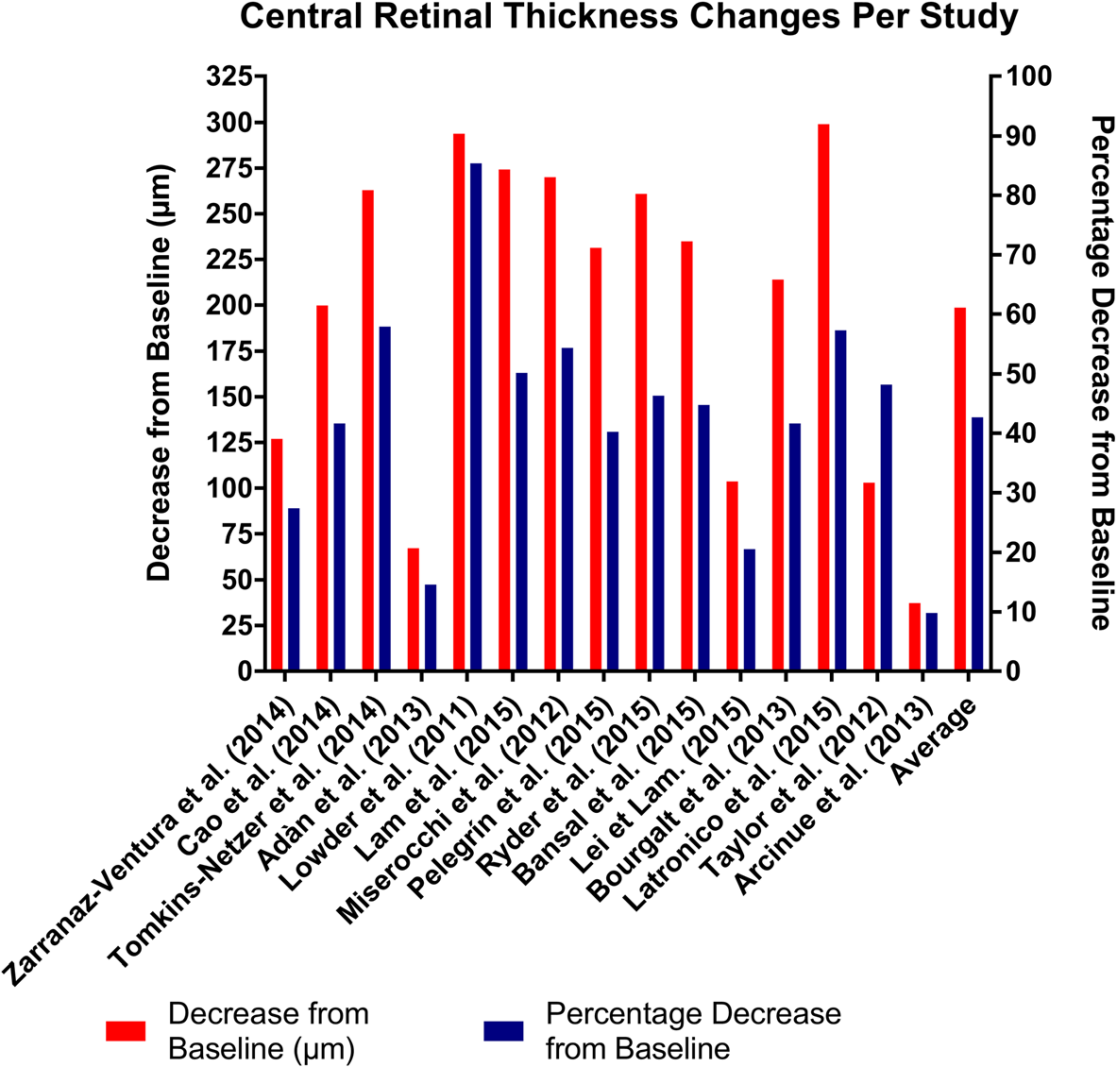
Central retinal thickness measurements:

Central retinal thickness was reduced from a range of 9.9 to 85.4% (Fig. 2) across all studies. However, most studies (80%) demonstrated a decrease of 20–60%. CRT decreased on average 42.7% from baseline, which equates to an average decrease of 198.65 μm (Additional file 1: Figure S1).

### Visual acuity

**Fig. 3:**
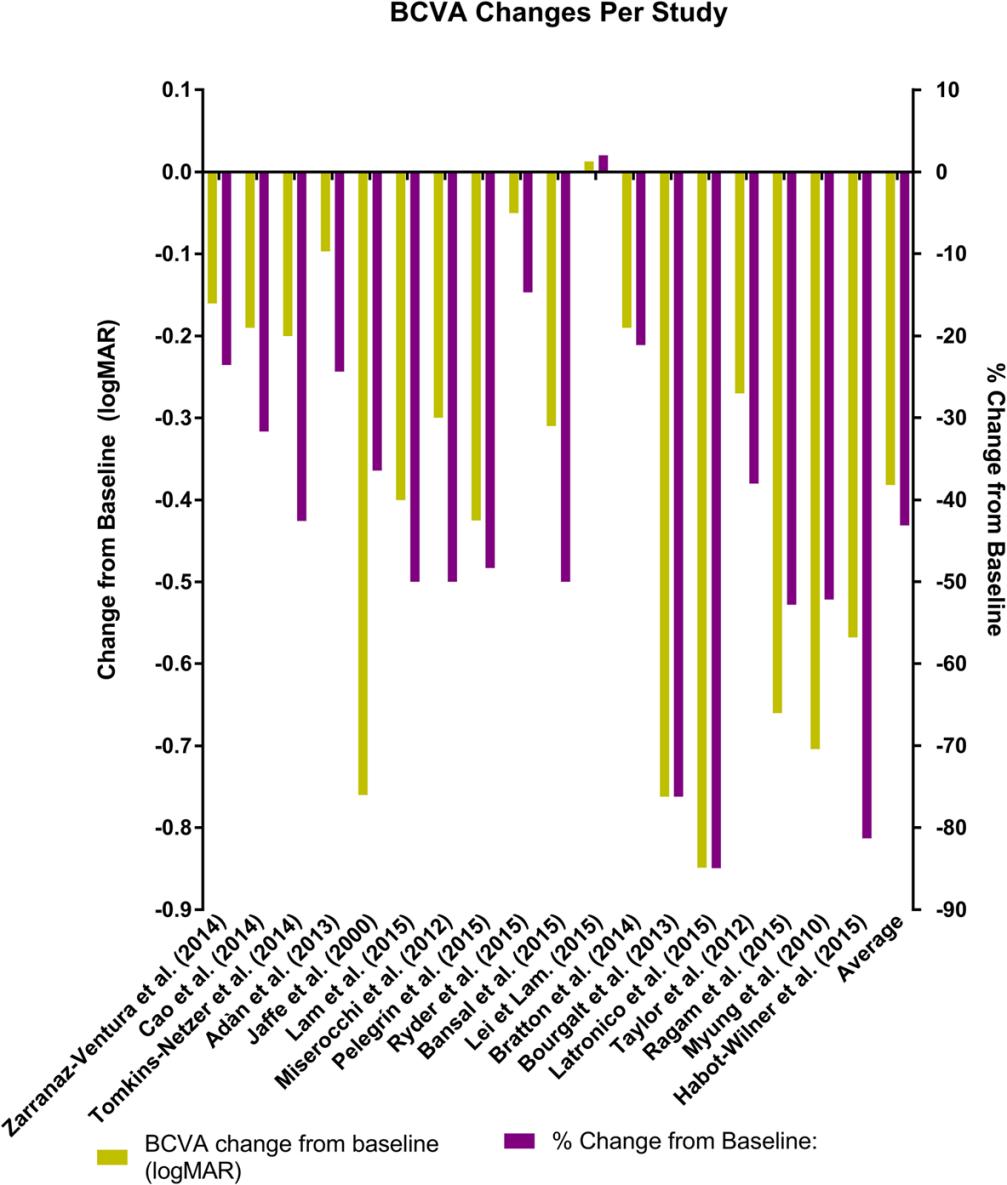
Best corrected visual acuity calculated values

Figure 3 shows the calculated values of visual acuity. The final best corrected visual acuity (BCVA) was logMAR 0.451 (Snellen BCVA of 20/57). Vision improved by 0.382 on the logMAR scale across all studies, which was a 43.1% improvement. Lowder et al. noted an improvement in average visual acuity in their trial, as patients treated with 0.7 mg DEX implant had an average improvement of 10.8 letters*.* Arcinue et al. [24] also found improved visual acuity in 18.2% of their patients treated with the DEX implant (Additional file 1: Figure S2).

**Table 1:** The numbers of quiescent eyes before and after the DEX implant

	Quiescent eyes before DEX implantation	Quiescent eyes after DEX Implantation	Eyes which became quiescent	Total eyes
Zarranz-Ventura et al. (2014) [6]	46	47	1	82
Cao et al. (2014) [7]	27	27	0	27
Tomkins-Netzer et al. (2014) [8]	22	20	− 2	38
Lowder et al. (2011) [1]	17	23	6	77
Jaffe et al. (2000) [10]	0	1	1	2
Pelegrín et al. (2015) [13]	21	38	17	42
Bratton et al. (2014) [17]	0	9	9	14
Bourgalt et al. (2013) [18]	0	0	0	2
Latronico et al. (2015) [19]	0	1	1	2
Myung et al. (2010) [22]	0	6	6	6
Habot-Wilner et al. (2015) [23]	0	1	1	1
Total	133	173	40	293

### Inflammatory markers

An initial 133 eyes (45.4%) were quiescent and 173 eyes (59%) were quiescent at the end of the studies. The DEX implant achieved quiescence in 40 eyes (13.7%) which previously had active inflammation (Table 1). Although Arcinue et al. provided no quantitative data, they found improvements in anterior chamber and vitreous inflammation. All studies showed an increase in the number of quiescent eyes with DEX implant use, except the study by Tomkins-Netzer et al. that had 2 eyes worsen (Table 1).

**Table 2:** Information about the selected studies

	Type of study	Population age group:	Final time point for data collection (months):
Zarranz-Ventura et al. [6]	Retrospective chart review	Adults	12
Cao et al. [7]	Retrospective chart review	Adults and Pediatric	3
Tomkins-Netzer et al. [8]	Retrospective chart review	Adults	24
Adán et al. [9]	Retrospective chart review	Adults	6
Lowder et al. [1]	Randomized control trial	Adults	6.5
Jaffe et al. [10]	Case report	Adults	30
Lam et al. [11]	Retrospective chart review	Adults	No set time point
Miserocchi et al. [12]	Retrospective chart review	Adults	No set time point
Pelegrín et al. [13]	Retrospective chart review	Adults	24
Ryder et al. [14]	Retrospective chart review	Adults	6
Bansal et al. [15]	Prospective interventional non-randomized study	Adults	6
Lei et Lam [16].	Case report	Pediatric	13
Bratton et al. [17]	Retrospective chart review	Pediatric	No set time point
Bourgalt et al. [18]	Case report	Pediatric	3
Latronico et al. [19]	Case report	Pediatric	1.25
Taylor et al. [20]	Retrospective chart review	Pediatric	6
Ragam et al. [21]	Retrospective chart review	Adults	6
Myung et al. [22]	Retrospective chart review	Adults and pediatric	2.6–6
Habot-Wilner et al. [23]	Case report	Adults	24
Arcinue et al. [24]	Retrospective chart review	Adults	24

### Adverse events

**Fig. 4:**
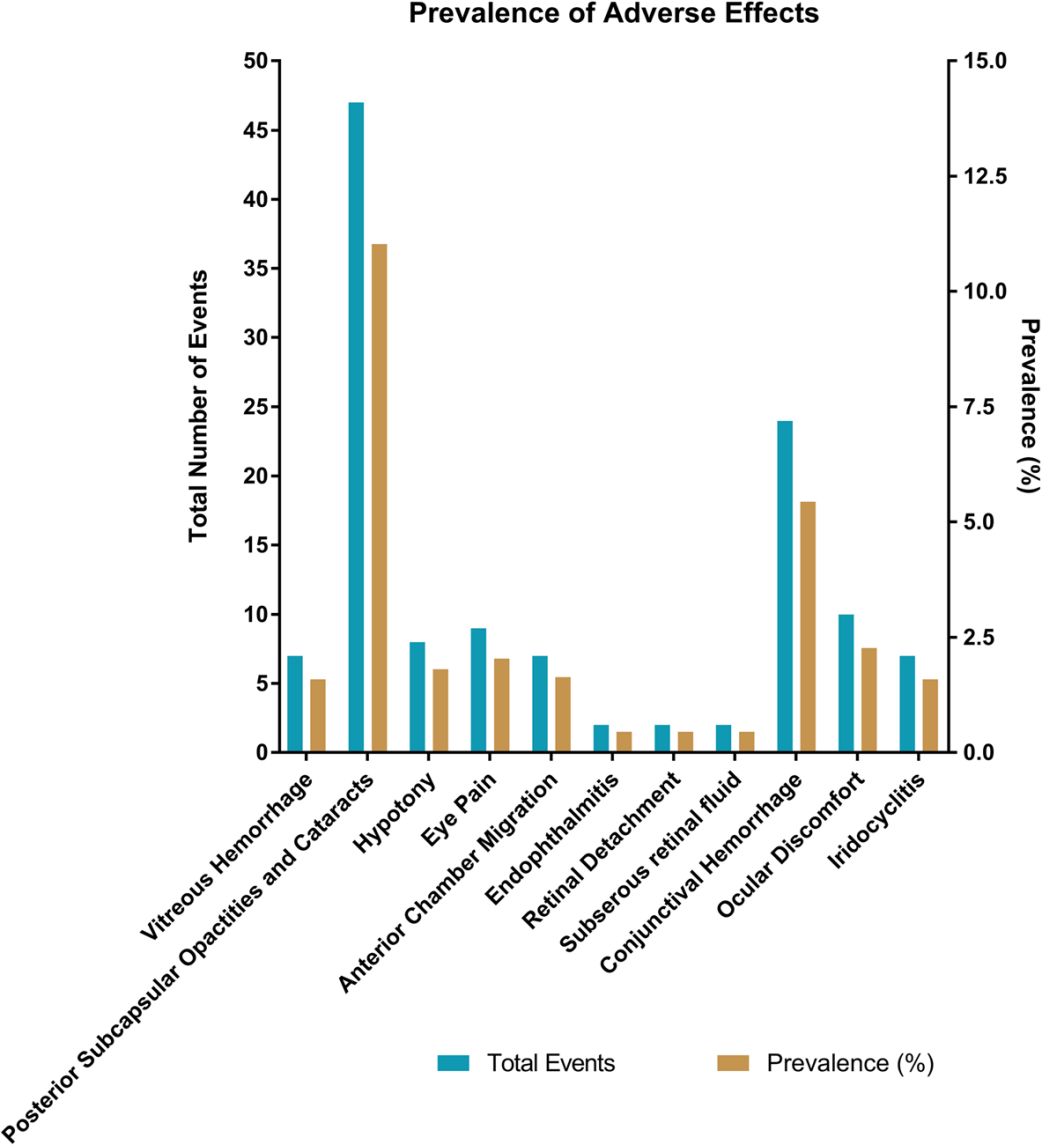
Prevalence of adverse events

Figure 4 summarizes adverse events across the 20 trials. There were two cases (0.5%) of endophthalmitis, retinal detachment, and macular edema. Iridocyclitis, anterior chamber migration, and vitreous hemorrhage occurred in seven cases (1.6%). Eight cases (1.8%) of hypotony occurred while nine patients (2.0%) had eye pain and 10 patients (2.3%) complained of ocular discomfort. The most common adverse events were subconjunctival hemorrhage and posterior subcapsular opacities and cataracts in 24 cases (5.4%) and 47 cases (11.0%), respectively. Bratton et al. documented DEX implant migration into the anterior chamber and the presence of posterior subcapsular opacities. Four studies (Cao et al., Bansal et al., Latronico et al., and Habot-Wilner et al.) found no adverse events (Additional file 1: Figure S3).

### Intraocular pressure

The total number of IOP adverse events in all 20 trials was 91 (20.6%) (Additional file 1: Figure S4). Four hundred forty-one eyes of 358 patients had intermediate, posterior, or panuveitis. One hundred ninety-five patients (61.5%) had previous treatments, including topical steroid, periocular injection, or other intravitreal corticosteroids before being treated with the DEX implant (Additional file 1: Figure S5). Two hundred thirty-two patients (64.8%) had been treated with oral corticosteroid and/or systemic immunosuppressants while simultaneously having the DEX implant (Additional file 1: Figure S6).

The average patient age was 39.7 (range 9–82 years old). As shown in Table 2, five of the 20 (25%) publications studied pediatric cases, 13 (65%) studied adult cases, and two (10%) papers studied a combination of adult and pediatric cases.

Thirteen cases were retrospective chart reviews, five were interventional case reports, one was a cohort study, and one was a randomized control trial.

Six of the studies ended treatment around 6 months, three studies had an endpoint earlier than 6 months (1.25 and 3 months), and seven studies had an endpoint more than 6 months (12–30 months) (Table 2). The paper by Myung et al. studied three cases with different time points ranging from 2.4–6 months while three of the papers had not set a time point but used the data at the end of treatment for each patient (which varied between patients) (Table 2).

## Discussion

The DEX implant is practical for treating uveitis and uveitic macular edema, and its ease of delivery and favorable risk profile is preferable compared to systemic corticosteroids. This literature review combines data from pediatric and adult cases to assess the effect of the DEX implant. Most of the data comes from retrospective reviews and are reliable sources when analyzed against the OCEMLEC [5]. The HURON trial (Lowder et al.) is the only RCT done with the DEX implant and its data mirrors the retrospective reviews. According to the OCEMLEC our study would rank at the “3a” level as we mostly combined data from case control studies [5].

### Central retinal thickness

The DEX implant almost unanimously reduced retinal thickness apart from the case reported by Arcinue et al.; the DEX implant was shown to have reduced macular thickness by an average of 42.7% (range 9.9 to 85.4%) from baseline across all studies. As cystoid macular edema is common sequelae of uveitis, the DEX implant is an effective option for treating posterior segment uveitis with macular edema.

### Visual acuity

Overall, the studies have shown that the DEX implant is effective at improving vision. Except for the study by Lei and Lam, there was a significant improvement in logMAR visual acuity, ranging from 21.1–85.0% improvement (Fig. 2). The study which showed deterioration in visual acuity only showed a mild decrease in visual acuity of 0.013 logMAR (2.1%). The HURON trial by Lowder et al. showed a 10.8 letter improvement in BCVA in the 0.7 mg the DEX implant group. The average improvement was 0.38 log scores or a 43.1% improvement from baseline.

### Inflammatory markers

After treatment, 40 eyes (13.7%) had an anterior chamber cell score of ≤ 0.5+ and/or vitreous haze ≤ 1+. In all the trials, except the review by Tomkins-Netzer et al., the DEX implant successfully reduced inflammation. Since the number of eyes achieving a score of 0.5+ and 0+ was almost always grouped together, it was not possible to demonstrate improvement in eyes from an anterior cell score of 0.5+ to 0+. The magnitude of effect of the DEX implant on reducing inflammation is undermined by this ceiling effect. The data shows the DEX implant to be effective in reducing the concomitant inflammation in noninfectious uveitis.

### Adverse events

Cataracts and PSCO were the most common adverse events occurring in 47 cases (11.0%). This is an expected finding, as local ocular steroid treatment results in cataract formation; however, it is not clear whether cataract was due to steroid treatment or associated with uveitis. Nonetheless, patients should be monitored for lens opacities. Subconjunctival hemorrhage was the second most common side effect occurring in 24 cases (5.4%).

A concern with intraocular implants and steroid usage is secondary glaucoma; our review confirmed this to be a tangible risk. A total of 102 eyes (23.1%) had elevated IOP; therefore, it is imperative to monitor the IOP of patients receiving the DEX implant. Endophthalmitis, retinal detachment, and subretinal fluid were very rare adverse events (0.5%). Implant migration to the anterior chamber occurred in seven cases (1.6%); most of these patients were aphakic (5/7 or 71.4%). The studies by Adan et al. and Ragam et al. do not specify whether their patients were aphakic or not. A study by Kang et al. found implant migration into the anterior chamber in four of their patients (0.4%) [25]. It is important to inform patients of this risk as corneal edema and severe visual disturbances can occur, or the implant could be deferred until after a secondary intraocular lens implant is placed in aphakic patients. Vitreous hemorrhage and hypotony were rare complications having a prevalence of 1.6% and 1.8%, respectively. Eye pain and ocular discomfort were relatively rare complications occurring at 8 (1.8%) and 10 cases (2.3%). This suggests that the DEX implant does not significantly impair a patient’s quality of life.

### Endophthalmitis and retinal detachments

The higher than expected prevalence of endophthalmitis and retinal detachments may be due to injection technique or the DEX implant. Because placing an intravitreal implant uses a similar techniques to injecting anti-VEGF medications, to determine whether complications are due to the intravitreal injection or due to the DEX implant, we looked at the prevalence rates of these complications in patients receiving anti-VEGF medications. The rates of endophthalmitis and retinal detachment were lower in anti-VEGF patients; in a meta-analysis by van der Reiss et al., endophthalmitis had an incidence rate of 0.09–0.11% and retinal detachment had an incidence of 0.01–0.08% [26]. The higher rates of endophthalmitis in the DEX implant patients could be explained by the fact that the 22-gauge (inner diameter 0.413 mm) DEX implantation device has a larger bore compared with the 30-gauge (inner diameter 0.159 mm) intraocular needles. This creates a larger needle tract in the globe which may have a higher probability of developing endophthalmitis.

### Conducting a pars plana vitrectomy and DEX implantation simultaneously

Following vitrectomy surgery, medications have a shorter half-life in the vitreous and patients may receive more frequent intravitreal injections. One of the studies analyzed the effectiveness of the DEX implant in vitrectomized and non-vitrectomized eyes. Vitrectomized eyes had a 49.1% reduction in CRT, whereas non-vitrectomized eyes had a 32.0% reduction in CRT [13]. Vitrectomized eyes had a greater improvement in visual acuity improving it by 71.6% compared to non-vitrectomized eyes at 62.1% [13]. Vitreous haze improvement was the same between both groups [13]. It is clear that vitrectomized eyes fair better outcomes compared with non-vitrectomized eyes; however, the improvement is only slight and non-vitrectomized eyes still have significantly favorable outcomes.

### The use of different endpoints

As Table 2 shows, there is variation in the duration of each of the studies. The DEX implant varies in effectiveness for different severities of uveitis. In the typical onset and course of uveitis, a single DEX implant begins working around 6–8 weeks and can last for about 6 months. In more severe uveitis, a single dose could have a lifetime of 4 months. Variable end points would explain the findings from Lowder et al. and Ragam et al. which had low IOP adverse events (7.3% and 0%, respectively) at their 6-month endpoints, while Latronico et al. found at the end of 5 weeks, 50% of their eyes had elevated IOP.

### Monotherapy or adjunct therapy

The DEX implant has been used as a monotherapy in clinical practice; however, there is currently no literature analyzing the benefits of the DEX implant in such a manner. In this review, 61.5% of all the study participants had previous immunomodulatory therapy (IMT) and 64.8% of all participants were treated with IMT and the DEX implant. Whether the DEX implant is effective as a monotherapy versus adjunctive therapy remains unknown and further research is needed.

### Future research

In our review, 8/20 studies (40%) did not report data on changes in inflammatory score despite the DEX implant’s indication for treating posterior uveitis. To date, there has been only one RCT (Lowder et al.) studying the DEX implant. More RCTs need to be conducted to investigate the DEX implant as an effective treatment. Although this systematic review was able to study the prevalence of ocular pain and discomfort, many other factors on quality of living that are still unknown. A useful tool to measure the quality of life with ocular mediations is the National Eye Institute Visual Functioning Questionnaire 25 (NEI VFQ-25). Further research measuring the NEI VFQ-25 score changes would shed more light onto the effects of the DEX implant and patient quality of life.

## Conclusion

The DEX implant is an effective medication for treating uveitis across all age groups as an adjunctive therapy to immunosuppressants. The DEX implant decreased central retinal thickness significantly, improved visual acuity and moderately improved inflammation. However, the DEX implant caused elevated intraocular pressure in a minority of cases, and therefore, it is important to monitor intraocular pressure over duration of effect of the DEX implant. Subconjunctival hemorrhage and posterior subcapsular cataract are possible adverse events. More RCTs are needed to increase the power of evidence supporting the use of the DEX implant. The effects of the DEX implant on quality of life are also unknown and need further exploration. Further research needs to be conducted to assess the DEX implant as a monotherapy for posterior uveitis.

## Additional file

**Additional file 1:** **Figure 1.** Changes in Central Retinal Thickness Across Studies. **Figure 2.** The Average Improvement in Visual Acuity (logMAR) per Study. **Figure 3.** The Prevalence of Adverse Effects. **Figure 4.** The Number of Eyes with an Adverse IOP Event per study. **Figure 5.** The Number of Eyes with Systemic Treatment before Ozurdex Implantation. **Figure 6.** The Number of Eyes with/without Systemic Treatment while being Treated with an Ozurdex Implant.

## Data Availability

All data generated or analysed during this study are included in this published article [and its supplementary information files.

## Abbreviations

AE's: Adverse Effects
BCVA: Best Corrected Visual Acuity
CRT: Central retinal thickness
DEX implant: Intravitreal dexamethasone implant
FDA: Food and Drug Administration
IMT: Immunomodulatory therapy
IOP: Intraocular pressure
NEI VFQ-25: National Eye Institute Visual Functioning Questionnaire 25
NICE: National Institute for Health and Care Excellence
OCEMLEC: Oxford Centre for Evidence-based Medicine Levels of Evidence criteria
OCT: Optical coherence tomography
PSCO: Posterior subcapsular opacities
RCT: Randomized controlled trial
