# Supplementary material for: Ozurdex (dexamethasone intravitreal implant) for the treatment of intermediate, posterior, and panuveitis: a systematic review of the current evidence
Source: J Ophthalmic Inflamm Infect. 2020 Jan 10;10:1. doi: 10.1186/s12348-019-0189-4 (PMC6954157; doi:10.1186/s12348-019-0189-4)
Supplement: Supplementary file 1 — Additional file 1: Figure 1. Changes in Central Retinal Thickness Across Studies. Figure 2. The Average Improvement in Visual Acuity (logMAR) per Study. Figure 3. The Prevalence of Adverse Effects. Figure 4. The Number of Eyes with an Adverse IOP Event per study. Figure 5. The Number of Eyes with Systemic Treatment before Ozurdex Implantation. Figure 6. The Number of Eyes with/without Systemic Treatment while being Treated with an Ozurdex Implant. [file 12348_2019_189_MOESM1_ESM.docx]

**Supplementary Material:**

**Figure 1: Changes in Central Retinal Thickness Across Studies**

| Central Retinal Thickness | Final CRT (µm) | Percentage change | Change from baseline (µm) |
| --- | --- | --- | --- |
| Zarranz ventura et al. | 335 | -27.49% | 127 |
| Cao et al. | 278.9 | -41.70% | 199.8 |
| Tomkins-Nertzer et al. | 190.29 | -58.02% | 263 |
| Adan et al. | 394.1 | -14.62% | 67.5 |
| Lowder et al. | 50.2 | -85.41% | 293.8 |
| Jaffe et al. | Not Available |  |  |
| Lam et al. | 272 | -50.21% | 274.3 |
| Misserochi et al. | 226 | -54.44% | 270 |
| Pelegrin et al. | 343.1 | -40.28% | 231.45 |
| Ryder et al. 2015 | 302.13 | -46.34% | 260.87 |
| Bansal et al. | 289.07 | -44.83% | 234.92 |
| Lei et Lam. 2015 | 399.33 | -20.61% | 103.6666667 |
| Bratton et al. | Not Available |  |  |
| Bourgalt et al. | 299.5 | -41.67% | 214 |
| Latronico et al. | 321 | -57.40% | 299 |
| Taylor et al. | 325 | -48.23% | 103 |
| Ragam et al. | Not Available |  |  |
| Myung et al. | Not Available |  |  |
| Habot Wilner et al. | Not Available |  |  |
| Arcuine et al. | 341.8 | -9.86% | 37.4 |
| Average Final Measurement: | 291.1613333 | -42.74% | 198.6471111 |

The breakdown of the average changes in CRT across each study.

**Figure 2: The Average Improvement in Visual Acuity (logMAR) per Study**

|  | % change | Change From Baseline (logMAR) | final BCVA (logMAR) |
| --- | --- | --- | --- |
| Zarranz ventura et al. | -23.53% | -0.16 | 0.52 |
| Cao et al. | -31.67% | -0.19 | 0.41 |
| Tomkins-Nertzer et al. | -42.55% | -0.2 | 0.27 |
| adan et al. | -24.35% | -0.09691 | 0.30103 |
| lowder et al. | Not Available | Not Available | Not Available |
| Jaffe et al. | -36.44% | -0.7625 | 1.3 |
| Lam et al. | -50% | -0.4 | 0.4 |
| Misserochi et al. | -50% | -0.3 | 0.3 |
| Pelegrin et al. | -48.30% | -0.425 | 0.455 |
| Ryder et al. 2015 | -14.71% | -0.05 | 0.29 |
| Bansal et al. | -50% | -0.31 | 0.31 |
| Lei et Lam. 2015 | 2.06% | 0.01333 | 0.66 |
| Bratton et al. | -21.11% | -0.19 | 0.71 |
| Bourgalt et al. | -76.20% | -0.7619545 | 0.2380455 |
| Latronico et al. | -84.95% | -0.849485 | 0.150515 |
| Taylor et al. | -38.03% | -0.27 | 0.44 |
| Ragam et al. | -52.80% | -0.66 | 0.59 |
| Myung et al. | -52.15% | -0.704 | 0.646 |
| Habot Wilner et al. | -81.31% | -0.568336 | 0.130334 |
| Arcuine et al. |  | Not Available | Not Available |
| Average Change: | -43.11% | -0.382491972 | 0.451162472 |

The breakdown of the average changes in visual acuity using the logMAR scale across each study.

**Figure 3: The Prevalence of Adverse Effects**

|  | total | possible |
| --- | --- | --- |
| Vitreous Haemorrhage | 7 | 441 |
| Posterior Subcapsular Opacities | 47 | 426 |
| Hypotony | 8 | 441 |
| Eye Pain | 9 | 441 |
| Anterior Chamber Migration | 7 | 426 |
| Endophthalmitis | 2 | 441 |
| Retinal Detachment | 2 | 441 |
| Subserous Retinal Fluid | 2 | 441 |
| Conjunctival Haemorrhage | 24 | 441 |
| Ocular Discomfort | 10 | 441 |
| Iridocyclitis | 7 | 441 |

The full breakdown of the total number of eyes with each adverse effect.

**Figure 4: The Number of Eyes with an Adverse IOP Event per study**

|  | Number of Eyes with an Adverse IOP Event | Out of How Many Possible |
| --- | --- | --- |
| Zarranz ventura et al. | 18 | 82 |
| Cao et al. | 6 | 27 |
| Tomkins-Nertzer et al. | 7 | 38 |
| adan et al. | 8 | 17 |
| lowder et al. | 6 | 82 |
| Jaffe et al. | 0 | 2 |
| Lam et al. | 4 | 23 |
| Misserochi et al. | 3 | 12 |
| Pelegrin et al. | 20 | 42 |
| Ryder et al. 2015 | 4 | 20 |
| Bansal et al. | 4 | 30 |
| Lei et Lam. 2015 | 0 | 3 |
| Bratton et al. | 5 | 15 |
| Bourgalt et al. | 0 | 2 |
| Latronico et al. | 1 | 2 |
| Taylor et al. | 4 | 14 |
| Ragam et al. | 0 | 12 |
| Myung et al. | 0 | 6 |
| Habot Wilner et al. | 0 | 1 |
| Arcuine et al. | 1 | 11 |
| total | 91 | 441 |

The breakdown of the number of eyes that had experienced adverse IOP events across each study.

**Figure 5: The Number of Eyes with Systemic Treatment before Ozurdex Implantation**

|  | Number with Previous Systemic Treatment | Out of How Many Possible |
| --- | --- | --- |
| Zarranz ventura et al. | 52 | 82 |
| Cao et al. | 27 | 27 |
| Tomkins-Nertzer et al. | 22 | 38 |
| adan et al. | 6 | 13 |
| lowder et al. | 20 | 77 |
| Jaffe et al. | 1 | 1 |
| Lam et al. | Not available | Not Available |
| Misserochi et al. | 12 | 12 |
| Pelegrin et al. | Not Available | Not Available |
| Ryder et al. 2015 | 8 | 10 |
| Bansal et al. | Not Available | Not Available |
| Lei et Lam. 2015 | 3 | 3 |
| Bratton et al. | 14 | 14 |
| Bourgalt et al. | 0 | 2 |
| Latronico et al. | 1 | 1 |
| Taylor et al. | 11 | 11 |
| Ragam et al. | 8 | 12 |
| Myung et al. | 0 | 4 |
| habot Wilner et al. | 1 | 1 |
| Arcuine et al. | 9 | 9 |
| total | 195 | 317 |

The breakdown of the number of eyes across each study that have had previous systemic immunomodulatory therapy before Ozurdex implantation.

**Figure 6: The Number of Eyes with/without Systemic Treatment while being Treated with an Ozurdex Implant**

|  | Number with Systemic Treatment | Out of How Many Possible: |
| --- | --- | --- |
| Zarranz ventura et al. | 34 | 63 |
| Cao et al. | 18 | 27 |
| Tomkins-Nertzer et al. | 20 | 27 |
| adan et al. | 11 | 13 |
| lowder et al. | 42 | 90 |
| Jaffe et al. | 1 | 1 |
| Lam et al. | 18 | 23 |
| Misserochi et al. | 12 | 12 |
| Pelegrin et al. | 19 | 32 |
| Ryder et al. 2015 | 6 | 10 |
| Bansal et al. | Not Available | Not Available |
| Lei et Lam. 2015 | 3 | 3 |
| Bratton et al. | 14 | 14 |
| Bourgalt et al. | 0 | 2 |
| Latronico et al. | 1 | 1 |
| Taylor et al. | 14 | 14 |
| Ragam et al. | 10 | 12 |
| Myung et al. | 0 | 4 |
| Habot Wilner et al. | 0 | 1 |
| Arcuine et al. | 9 | 9 |
| total: | 232 | 358 |

The breakdown of the number of eyes that had concomitant systemic treatment with Ozurdex across each study.
